# Cancer histone H2A.Z missense mutations disrupt function through distinct local and allosteric effects

**DOI:** 10.21203/rs.3.rs-8484515/v1

**Published:** 2026-01-12

**Authors:** Maria Aristizabal

**Affiliations:** Queens University

**Keywords:** H2A.Z, oncohistone, chromatin organization, nucleosome stability, nucleosome dynamics, allostery

## Abstract

H2A.Z is a conserved variant of histone H2A that functions in a wide range of processes including transcriptional, chromatin organization, and genome stability, but whose exact role in the cell remains incompletely understood. A growing body of literature highlights the utility of cancer histone missense mutations in revealing fundamental aspects of histone structure and function not captured by previous efforts including comprehensive alanine scans. Motivated by this work, we systematically examined the impact of cancer missense mutations affecting H2A.Z using *Saccharomyces cerevisiae* as a model system. This work led us to identify amino acids critical for normal H2A.Z function and the mechanisms by which mutations at those sites disrupt H2A.Z activity. Combining approaches from the fields of genetics, molecular biology, biochemistry, and biophysics, we found that cancer H2A.Z mutations show decreased genome-wide occupancy, disrupt interactions with DNA, other histones, and nucleosome-binding proteins, and decrease nucleosome stability. Importantly, we show that these effects are recapitulated in human cell lines carrying histone H2A.Z mutations, highlighting yeast as a powerful system to begin to understand the impact of cancer histone mutations. Collectively, our work identified previously unappreciated amino acids that are critical for normal H2A.Z function, revealing functional consequences of altered nucleosome structure and dynamics.

## Introduction

In eukaryotes, genetic information is organized into chromatin, a nucleoprotein structure that controls access to the underlying DNA sequence and by doing so regulates fundamental processes like transcription, DNA replication, and DNA repair. Chromatin is composed of nucleosomes, which are made up of ~ 147 bp of DNA wrapped around an octamer of histone proteins, typically two copies each of H2A, H2B, H3, and H4[1]. Nucleosomes are dynamic and can be modified by several mechanisms, including the addition of post-translational modifications (PTMs) to histone proteins; the eviction, deposition, and sliding of nucleosomes by chromatin remodelers; and the substitution of replication-dependent histones (also known as canonical histones) by histone variants[2–4].

Histone variants differ from replication-dependent histones in regulation, amino acid sequence, structure, and genomic distribution. As their name suggests, replication-dependent histones are primarily expressed during the S phase of the cell cycle and supply the bulk of histones needed to package newly synthesized DNA[5]. In contrast, histone variants are expressed throughout the cell cycle and their incorporation into chromatin is independent of DNA replication[6, 7]. Beyond these regulatory differences, the sequence and structure of histone variants enable them to interact with specialized chaperones and deposition complexes, which target them to specific genomic locations[7–10]. At these sites, histone variants modulate chromatin by altering local PTM patterns, recruiting distinct chromatin-associated proteins, and influencing nucleosome stability and dynamics[7, 9, 11, 12].

Histone H2A.Z is a highly conserved variant of H2A that functions in most DNA-dependent processes, including transcription, DNA replication and repair, chromosome segregation, and the maintenance of heterochromatin-euchromatin boundaries[13–15]. In vertebrates, H2A.Z is encoded by two genes (*H2AFZ* and *H2AFV*) that produce two main proteins, H2A.Z.1 and H2A.Z.2, respectively, that differ by only three amino acids[16]. p400 or SRCAP in human cells and Swr1 in yeast are ATP-dependent chromatin remodelers that incorporate H2A.Z into specific genomic regions, including the + 1 nucleosome of most gene promoters and sites of DNA damage[17–24]. H2A.Z exhibits structural and functional differences from H2A, sharing only approximately 60% sequence homology[25]. Key distinguishing features of H2A.Z include an extended acidic patch and conformational variations in the L1 loop[26, 27]. The latter localizes to the H2A-H2B dimer interface of the nucleosome and is thought to contribute to the decreased stability observed in H2A.Z-containing nucleosomes compared to those containing H2A[8, 11, 12]. Another region of H2A.Z that differs from H2A is the M6 segment, which serves as an interaction site for the H2A.Z deposition machinery[28–31].

Highlighting a key role for H2A.Z in cellular function, its overexpression is observed in a wide variety of cancers where it correlates with patient health outcomes[32–34]. Beyond overexpression, missense mutations affecting H2A.Z have also been detected in cancer patients[35]. The R80C mutation, the only H2A.Z cancer mutation characterized in detail to date, was shown to destabilize H2A.Z-containing nucleosomes *in vitro*, revealing a previously unknown role for the R80 residue in nucleosome stability[36]. As such, the study of cancer-associated histone mutations, alterations that are emerging as important contributors to cancer biology, provides new opportunities to understand normal histone function[37, 38]. Efforts examining the best characterized cancer histone mutations, H3K27M, H3K36M, H3G34R/V, and H2BE76K, revealed defects in PTMs, nucleosome-binding factor recruitment dynamics, nucleosome stability, and gene expression[39–42].

In this study, we leverage cancer-associated mutations to probe H2A.Z biology. Using *Saccharomyces cerevisiae* as a model system, we performed the most comprehensive analysis of H2A.Z mutations identified in cancer to date. Using a combination of *in vivo*, *in vitro*, and *in silico* approaches, we show that cancer H2A.Z mutations decrease genome-wide occupancy through diverse mechanisms that include reducing nucleosome stability and disrupting interactions with DNA, other histones, and H2A.Z-binding proteins. Highlighting a conserved effect, occupancy and nucleosome-binding protein defects detected in yeast were also observed in human cell lines. As such, our results reveal new amino acids that are important for normal H2A.Z activity while also highlighting mechanisms by which H2A.Z cancer mutations disrupt function, information critical to begin to understand how they contribute to oncogenesis.

## Results

### H2A.Z mutations affected most regions of the H2A.Z protein sequence

**Figure 1:**
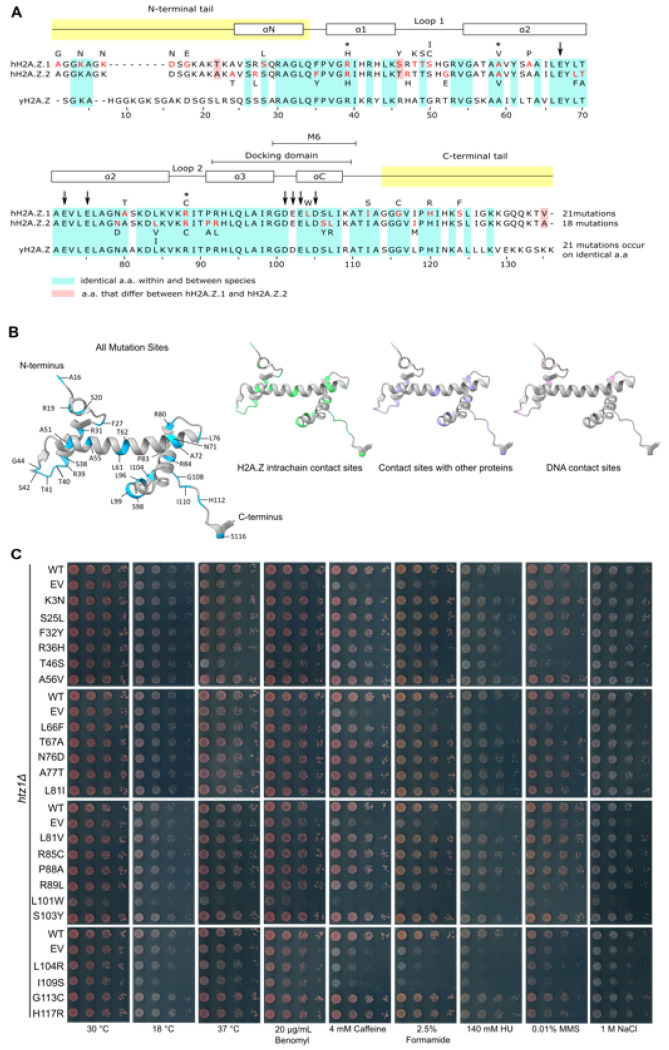
Distribution of H2A.Z missense mutations and their functional consequences **(A)** Protein sequence alignment of yeast (yH2A.Z) and human H2A.Z isoforms H2A.Z.1 (hH2A.Z.1) and H2A.Z.2 (hH2A.Z.2). Amino acids affected by missense mutations are shown in red text. For each mutation, the mutant amino acid is shown above the alignment if it occurs on hH2A.Z.1 and below if it occurs on hH2A.Z.2. Asterisks show mutations affecting both human H2A.Z isoforms. Structural and functional features of H2A.Z are shown above the sequence alignment. Acidic patch residues are shown with black arrows. Residues that are identical across species are highlighted in teal, and amino acids that differ between hH2A.Z.1 and hH2A.Z.2 are marked in pink. **(B)** Distribution of cancer-associated H2A.Z missense mutations mapped onto the H2A.Z protein structure (based on PDB 1F66). Blue residues indicate all mutation sites. Green residues denote mutation sites that also form internal H2A.Z contacts. Purple residues mark mutation sites that interact with other proteins. Pink residues highlight mutation sites that contact DNA. **(C)** The R36H, T46S, L66F, L101W, L104R, and I109S H2A.Z mutations produced a variety of growth defects in yeast cells. Representative image from three biological replicates showing 10-fold serial dilutions of *htz1Δ* strains carrying the indicated plasmids. *S*trains carrying the wild-type plasmid and empty vector (EV) serve as positive and negative controls, respectively.

Using whole exome sequencing data from over 13,000 cancer patients, we previously reported and computationally characterized > 2,800 unique cancer histone missense mutations that map to most histone genes and proteins[35, 43]. This dataset included 39 unique missense mutations in genes that code for H2A.Z: 21 mutations (54%) mapping to *H2AFZ* which codes for H2A.Z.1 and 18 mutations (46%) on *H2AFV* which codes for H2A.Z.2 (Fig. 1A and 1B; Supplementary Table S1). Cancer missense mutations mapped along the entire H2A.Z protein sequence and structure although the L1 loop was significantly enriched for mutated residues (6/9 or 67% of the amino acids were mutated - Fisher’s exact test p value 0.0238) when compared to the rest of the protein sequence. No other regions of the protein were enriched for mutated residues, although a cluster of mutations at the alpha C helix (3/6 or 50% of amino acids) was also observed. No mutations were detected on acidic patch residues (Fig. 1A; Supplementary Table S2), important contact sites for nucleosome-binding proteins[30, 31, 44]. Most H2A.Z mutations generated single amino acid substitutions at each position; the only exceptions were S42 and A81, which were mutated to I/C and I/V, respectively. Most mutations were also unique to each H2A.Z gene. R31H, A51V, and R80C were the only mutations observed on both H2A.Z.1 and H2A.Z.2 (Supplementary Table S1). Of these, the H2A.Z.1 R80C mutation is the only H2A.Z cancer mutation characterized to date, shown to localize to nuclei and associate with chromosomes *in vivo* despite destabilizing the nucleosome *in vitro*[36]. However, whether other cancer histone mutations disrupt normal H2A.Z function remains unknown.

#### Several histone missense mutations disrupted normal H2A.Z function

Several H2A.Z cancer mutations map to amino acids previously shown to be important for function[25, 45–49], raising the possibility that they may affect normal H2A.Z activity. Furthermore, because most cancer mutations replace wild-type residues with amino acids other than alanine, these mutations may disrupt H2A.Z beyond the effects captured by a previous comprehensive alanine scan[47]. To investigate the functional impact of cancer H2A.Z mutations *in vivo*, we leveraged the relative simplicity of the budding yeast model system, where H2A.Z is encoded by a single non-essential gene, *HTZ1*[16, 50, 51]. Importantly, H2A.Z is one of the most evolutionarily conserved histones across eukaryotic species, showing 74% sequence identity and 85% similarity between yeast and humans. The high level of identity allowed us to test the impact of 21 cancer histone H2A.Z mutations (Fig. 1A and 1C). As a conservative approach, mutations mapping to amino acids that are not identical between yeast and humans were excluded from this analysis.

To generate yeast cells carrying wild-type or mutant versions of *HTZ1* that mimic alterations observed in cancer patients, low-copy expression vectors were engineered to carry wild-type or mutant *HTZ1* driven by its endogenous promoter and fused to a C-terminal 3xFLAG epitope tag. These plasmids were then introduced into yeast cells lacking endogenous *HTZ1*. For simplicity, we refer to all *HTZ1* mutants by the resulting missense mutation in the yeast H2A.Z amino acid sequence and omit the *FLAG* label in all the figures. To identify mutations that affect normal H2A.Z function, the growth of yeast strains carrying wild-type or mutated *HTZ1* was examined in a wide range of conditions, including several where *HTZ1* was shown to be required for normal growth[17, 25, 46, 48, 52–55]. Consistent with previous reports, the *htz1Δ* mutant carrying an empty vector exhibited sensitivity across most conditions when compared to wild type (Fig. 1C; Supplementary Figure S1A). Although most strains carrying H2A.Z mutations did not show obvious growth defects, strains carrying the R36H (R31H in humans), T46S (T41S in humans), L66F (L61F in humans), L101W (L96W in humans), L104R (L99R in humans), and I109S (I104S in humans) alleles showed altered growth in specific conditions indicating that the resulting missense mutations alter H2A.Z activity (Fig. 1C; Supplementary Figure S1A). Intriguingly, no growth defects were observed for the R85C mutant (Fig. 1C), the yeast equivalent of R80C, despite this mutation being known to decrease nucleosome stability[36]. Focusing on mutants producing growth defects, the L66F mutant showed the mildest effects and only produced a slight growth decrease when exposed to methyl methanesulfonate (MMS). The R36H and T46S mutants had intermediate phenotypes and showed similar growth defects to one another but generally different from the *htz1Δ* mutant when exposed to caffeine, formamide, hydroxyurea (HU), and MMS. Beyond these phenotypes, the T46S mutant also showed growth defects at 37°C, effects that were much more severe than the *htz1Δ* mutant and not seen for the R36H mutant. The L101W, L104R, and I109S mutants produced the most widespread and severe growth defects. The L104R and I109S mutants showed similar growth defects when exposed to 37°C, benomyl, caffeine, HU, MMS, and osmotic stress (NaCl), phenotypes that resembled the *htz1Δ* mutant. Both mutants also showed growth defects when exposed to 18°C and formamide, although under these conditions, the I109S mutant phenotype was more severe than both the L104R and *htz1Δ* mutants. Finally, the L101W mutant showed growth defects that were more severe than the *htz1Δ* mutant under all conditions tested, including normal growth conditions (Fig. 1C; Supplementary Figure S1A). By in large, the mutant phenotypes were distinct from the *htz1Δ* strain indicating that their effects did not simply reflect a loss of *HTZ1*. Consistently, all six mutants showed bulk H2A.Z protein levels similar to the wild-type control (Supplementary Figure S1B). Here, the *htz1Δ* strain carrying an empty vector served as a negative antibody control. As such, despite previous studies highlighting I109 as critical for H2A.Z function[46, 47], how the other amino acids contribute to H2A.Z activity is unknown.

### H2A.Z mutations altered chromatin association

**Figure 2:**
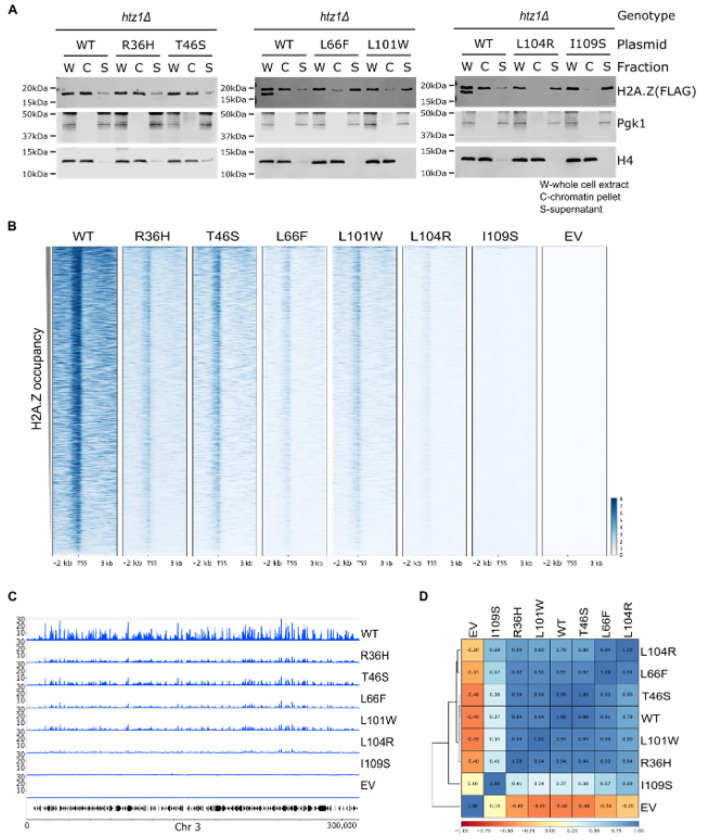
H2A.Z mutations alteredchromatin association and reducedgenome-wide occupancy **(A)** The L66F, L101W, L104R, and I109S mutations showed altered bulk chromatin association. Whole-cell extracts (W) were separated into chromatin (C) and supernatant (S) (non-chromatin) fractions and analyzed by immunoblotting. FLAG antibodies detected wild-type or mutant H2A.Z. H4 and Pgk1 were used as cellular fractionation controls and were primarily associated with the chromatin and supernatant fractions, respectively. **(B)** Heatmaps showing wild-type or mutant spike-in normalized H2A.Z occupancy at the transcription start sites (TSSs) of 6,021protein-coding genes. All heatmaps were sorted based on the H2A.Z signal intensity in the wild-type control. **(C)** Representative genome browser track showing spike-in normalized H2A.Z occupancy across a region of chromosome (Chr)III. The Y-axis represents normalized read density (mean counts per 10 bp). **(D)** Pearson correlation coefficients of ChIP-seq signal profiles binned at 100 bp resolution.

Having ruled out changes to total H2A.Z protein levels, we next assessed whether the mutations altered the levels of H2A.Z in chromatin using bulk cellular fractionation assays. As reported previously, wild-type H2A.Z preferentially localizes to the chromatin fraction, although a small amount is clearly visible in the non-chromatin-bound fraction[25, 48]. Focusing on the mutants, R36H and T46S showed normal levels of H2A.Z chromatin association. By contrast, the L66F and L101W mutants exhibited reduced, but still detectable, chromatin-bound H2A.Z, whereas the L104R and I109S mutants showed little to no H2A.Z in chromatin (Fig. 2A), findings indicative of defects in H2A.Z incorporation, retention, or both. To determine if altered chromatin association is a widespread effect of cancer H2A.Z mutations, we extended the cellular fractionation assay to the S25L, L81V, G113C, and R85C mutants, capturing mutations along the length of the H2A.Z protein as well as mutations previously shown to decrease nucleosome stability[36]. The S25L, L81V, and G113C mutants showed normal levels of H2A.Z in the chromatin and non-chromatin fractions. By contrast, the R85C mutant showed reduced H2A.Z levels in chromatin and elevated levels in the non-chromatin-bound fraction (Supplementary Figure S2), findings consistent with previous reports[36].

### H2A.Z mutations decreased occupancy genome-wide

Having observed bulk changes in H2A.Z chromatin association in several mutants, we next asked if the defects reflected alterations at specific loci or across the genome. To this end, we performed chromatin immunoprecipitation followed by high-throughput sequencing (ChIP-seq) for H2A.Z using anti-FLAG antibodies. To ensure that the signal detected was specific for H2A.Z, the *htz1Δ* strain carrying an empty vector control was profiled. To detect global changes in H2A.Z occupancy, *Schizosaccharomyces pombe* cells carrying *RPB3-FLAG* were spiked into every sample before cell lysis, allowing us to capture all sources of variability throughout the ChIP protocol. Details about *S. cerevisiae* and spike-in read counts can be found in Supplementary Tables S3 and S4. ChIP-seq experiments were performed in biological triplicate, and both input and IP samples were sequenced. Overall, Pearson correlation coefficients were > 0.89 across biological replicates (Supplementary Figure S3), indicating high levels of reproducibility. The only exceptions were the empty vector control and the I109S mutant, which showed Pearson correlation coefficients lower than 0.80 but greater than 0.66 indicating higher levels of variability (Supplementary Figure S3).

Consistent with previous reports, H2A.Z localized to transcriptional start sites (TSSs)[19, 20, 49, 56–59], and no signal was detected in the *htz1Δ* empty vector control (Fig. 2B). Following spike-in normalization, all mutants showed reduced H2A.Z occupancy at TSSs, with the strongest effect observed for the I109S mutant, followed by the L104R, L66F, R36H, L101W, and T46S mutants (Fig. 2B). Consistently, only 27 peaks were detected in the I109S mutant compared to 2,914 H2A.Z peaks in wild type; 2,582 in L101W; 2,472 in T46S; 2,315 in R36H; 2,090 in L66F; and 1,220 in L104R (Supplementary Table S5). Importantly, most peaks identified in the wild type were also detected in the mutants, with only a small number of peaks detected only in the mutants (Supplementary Figure S4). In line with these findings, genome-wide H2A.Z occupancy profiles were largely similar across the mutants and between the mutants and wild type (Fig. 2C). Pearson correlation coefficients exceeded 0.79 for all pairwise comparisons, except for the I109S mutant, which showed almost no detectable H2A.Z occupancy across the genome and had Pearson correlation coefficients < 0.69 when compared to wild type and the other H2A.Z mutants (Fig. 2D). Collectively, these findings suggest that the mutant versions of H2A.Z retain normal deposition patterns, suggesting that defects in stability rather than deposition may underscore the observed occupancy defects. In support of this hypothesis, the H2A.Z mutant growth phenotypes were distinct from the *swr1Δ* mutant (Supplementary Figure S5), which removes the catalytic subunit of the SWR1 complex and completely blocks H2A.Z deposition[17, 54]. As reported previously, *swr1Δ* mutants show growth defects that are less pronounced than *htz1Δ* mutants, with *htz1Δ swr1Δ* double mutants phenocopying *swr1Δ*[17, 49, 54, 60, 61]. More specifically, the R36H, T46S, and L66F mutants showed phenotypes that were milder than the *swr1Δ* mutant when exposed to caffeine, while the L101W, L104R, and L109S mutants showed stronger phenotypes than the *swr1Δ* mutant, effects clearly observed when strains were exposed to caffeine, formamide, and HU. Importantly, combining the H2A.Z mutations with the *swr1Δ* allele resulted in *swr1Δ*-like phenotypes indicating that the effect of the H2A.Z mutants depended on their ability to be incorporated into chromatin.

#### H2A.Z mutations differentially affected HTZ1-dependent gene expression

**Figure 3:**
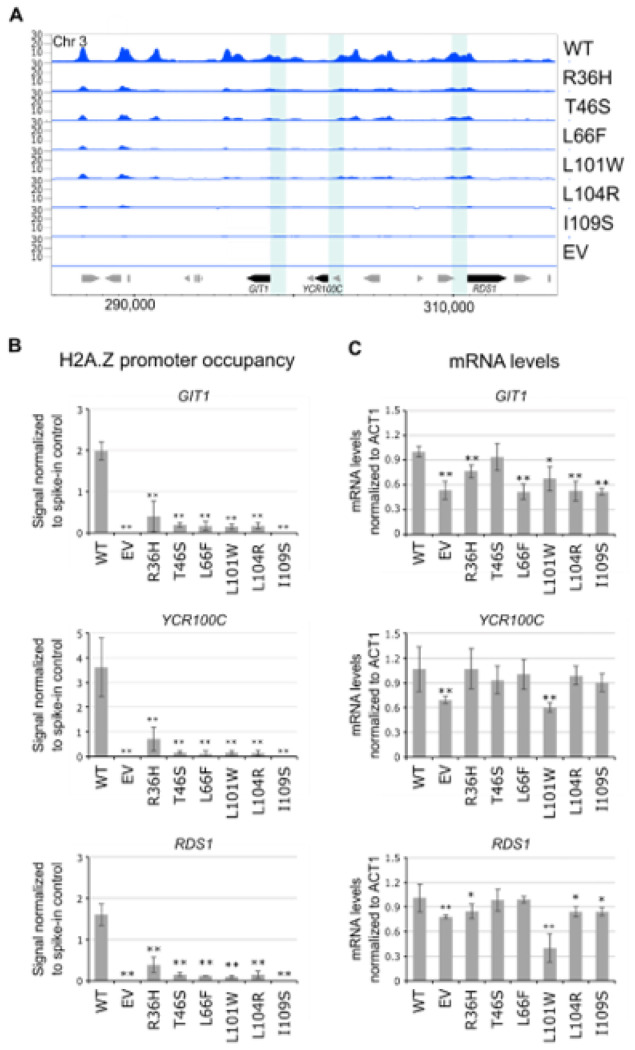
H2A.Z mutations differentially affectedtranscription **(A)** Genome tracks showing H2A.Z spike-in normalized ChIP-seq signal at a subset of H2A.Z-dependent genes located between the *HMRa* locus and the right telomere of chromosome III. The Y-axis values indicate the mean normalized reads per 10 bp. H2A.Z-dependent genes, *GIT1*, *YCR100C*, and *RDS1* are shown at the bottom of the figure, and their promoters are highlighted in teal boxes, respectively. (**B)** H2A.Z occupancy at the indicated *HTZ1*-dependent genes (*GIT1*, *YCR100C*, and *RDS1*) as determined by ChIP-qPCR. Signal intensities were normalized to spike-in controls. Bars represent the mean of three biological replicates with error bars showing standard deviation. Statistical significance was assessed using an unpaired two-tailed Student’s *t*-test. P-values are denoted as follows: **P < 0.01. (**C)** mRNA levels of *HTZ1*-dependent gene (*GIT1*, *YCR100C*, and *RDS1*) measured via RT-qPCR. mRNA levels were normalized to *ACT1*. Bars represent the mean of three biological replicates with error bars showing standard deviations. Statistical significance was assessed using unpaired two-tailed Student’s *t*-tests. p-values are denoted as follows: *P < 0.05, **P < 0.01.

Finding that several mutations decreased H2A.Z occupancy led us to examine whether they also affected *HTZ1*-dependent gene expression. To test this possibility, we focused on *GIT1*, *YCR100C*, and *RDS1*, genes previously reported to depend on *HTZ1* for normal mRNA levels and that exhibited decreased H2A.Z occupancy in our ChIP-seq profiles (Fig. 3A)[25, 48, 55]. As a negative control, we examined *ALD5*, a gene not known to depend on *HTZ1* for gene expression, and that showed minimal H2A.Z occupancy in the wild type (Supplementary Figure S6). As expected, no changes in *ALD5* mRNA levels were observed in any of the mutants (Supplementary Figure S6). Importantly, we validated the decrease in H2A.Z occupancy at the *GIT1*, *YCR100C*, and *RDS1* gene promoters by ChIP-qPCR (Fig. 3B). Focusing on gene expression, we found that all mutants except for T46S significantly decreased *GIT1* mRNA levels when compared to wild type (Fig. 3C). For *RDS1*, most mutants also significantly decreased mRNA levels except for the T46S and L66F mutants (Fig. 3C). These results were surprising, given that the L66F mutant showed less H2A.Z occupancy at the *RDS1* promoter compared to the R36H and L101W mutants which decreased *RDS1* mRNA levels. *YCR100C* mRNA levels were only reduced in the L101W mutant, although many other mutants like L66F, L104R, and I109S showed less H2A.Z occupancy at the *YCR100C* promoter and did not show altered *YCR100C* expression. Collectively, these findings demonstrate that while H2A.Z mutations do disrupt *HTZ1*-dependent gene expression, their effects are gene-specific and not strictly correlated with H2A.Z levels at gene promoters, findings consistent with the enigmatic role of H2A.Z in transcription[14, 25, 49, 62].

### H2A.Z mutations were predicted to induce local and long-range allosteric effects

**Figure 4:**
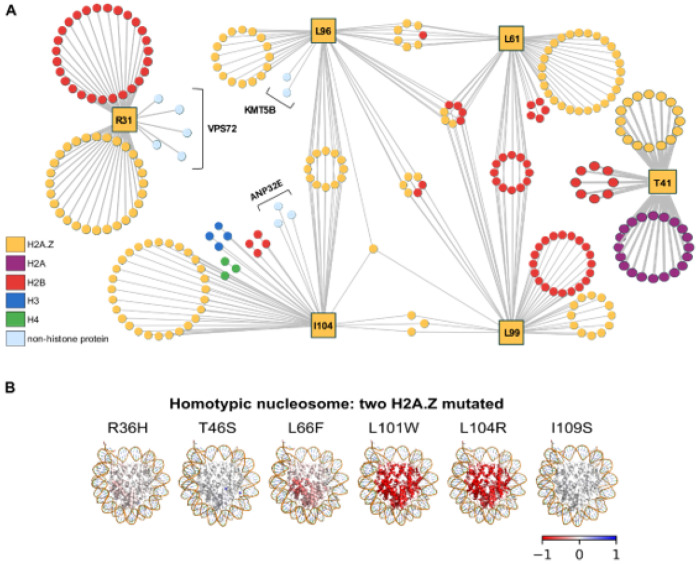
H2A.Z mutations are predicted to disrupt physical interactions via proximal and long-range allosteric effects **(A**) Subgraphs showing amino acid level contacts for H2A.Z residues R31, T41, L61, L96, L99, and I104 (yellow squared nodes). Amino acids on H2A.Z, other histones, or non-histone proteins that physically contact the mutated residues (yellow square nodes) are shown as circular nodes. The color of the circular nodes corresponds to the type of protein the residues belong to. For simplicity edges connecting circular nodes are not shown. Networks were visualized using Cytoscape[136]. **(B**) Nucleosome structures depicting per-residue average allosteric modulation (Δh) effect induced by the indicated cancer mutations. Mutations were introduced into both copies of H2A.Z. Values of |Δh| ≥ 0.6 kcal/mol are considered significant. Structures are based on PDB 5B33. Allosteric effects were calculated using PDB 5B33, PDB 3WA9, PDB 3WAA, and PDB 1F66 for the homotypic H2A.Z nucleosome structures.

We recently reported that histone mutations are predicted to disrupt interactions with other histones and nucleosome-binding proteins[35]. To determine whether H2A.Z mutations may have similar effects, we projected each mutated site onto an experimentally determined histone-centric physical interactome constructed from human histone complexes (see methods for details on network construction). For each of the six mutated H2A.Z residues, we retrieved all direct contacts at the residue level that met our distance criteria (Fig. 4A). This analysis revealed overlapping interaction networks for the L61, L96, L99, and I104 residues. Briefly, the L61, L96, and L99 residues shared contacts with H2B while the L99, L96, and I104 residues shared intrachain contacts within H2A.Z. These findings are consistent with these amino acids localizing to the same region of H2A.Z (Fig. 1B) and suggest that mutations at these sites may produce shared effects on protein-protein interactions and ultimately function. Nevertheless, the network analysis revealed clear differences. L96 (equivalent to yeast L101) was the only residue to contact KMT5B, a histone methyltransferase that modifies L20 on histone H4[63]. I104 (equivalent to yeast I109) made unique contacts with H3, H4, and AN32E, a histone chaperone involved in the removal of H2A.Z from the nucleosome[64, 65]. R31 (the human equivalent of R36) made unique contacts with residues on VPS72, a subunit of the SWR1 complex responsible for depositing H2A.Z into chromatin[17, 28]. T41 (equivalent to T46 in yeast) was the only residue that made contacts with H2A, findings consistent with this amino acid sitting at the nucleosome dimer interface[12]. Collectively, these results suggest that mutations at these positions may disrupt function through partially overlapping but generally distinct mechanisms, effects consistent with differing effects on yeast growth and chromatin occupancy. Furthermore, this analysis points to specific activities that may be altered by some of the mutations, including H2A.Z deposition and H4K20 methylation.

We previously showed that cancer histone mutations may disrupt interactions beyond the mutated site via long-range allosteric effects on the nucleosome[66]. To determine if this may also be the case for H2A.Z mutations, we used AlloSigMA 2[67] to calculate per-residue allosteric free-energy changes (Δh) between mutant and wild-type nucleosomes. Each mutation was assessed across all available H2A.Z nucleosome structures: four homotypic H2A.Z (PDBs: 5B33, 3WA9, 3WAA, and 1F66) and two heterotypic H2A/H2A.Z structures (PDBs: 5B31 and 5B32) as both states occur *in vivo*[68–70]. In homotypic structures, we also modelled the effect of one or both copies of H2A.Z carrying the cancer mutation, given that cancer cells typically carry both wild-type and mutant H2A.Z proteins[71]. Allosteric modulation was evaluated by calculating the change in free energy between the unperturbed (wild type) and perturbed (mutant) states with respect to the background of the whole protein (Δh). A negative Δh value indicates stabilization of the region, while a positive value indicates destabilization. Regardless of which structure was used and whether one or two mutations were introduced, mutating the R36H, T46S, I109S, and L66F equivalent positions did not show strong allosteric effects, defined as |Δh| ≥ 0.6 kcal/mol at sites located at least 11 Å away from the mutated site (Fig. 4B; Supplementary Figure S7) [72]. Nevertheless, a trend towards decreased allosteric modulation (stabilization) was observed for the L66F mutation in all types of nucleosome structures, regardless of whether one or two mutations were modelled (Fig. 4B; Supplementary Figure S7). In fact, an interesting pattern emerged. In homotypic nucleosomes, when both copies of H2A.Z were mutated, the allosteric effect was seen for both copies of H2B but when only one copy was mutated, the effect only extended to the H2B molecule closest to the mutated histone, indicating spatial specificity in the propagation of allosteric effects. The L101W and the L104R mutations showed strong allosteric effects along the alpha 2 and 3 helices of H2A.Z that extended to the alpha 2 and C helices on the closest H2B and the C-terminus of the closest H3 molecule in heterotypic nucleosomes (Supplementary Figure S8A and S8B). A similar trend was observed in structures of homotypic nucleosomes with one or two L101W or L104R mutations, but these did not pass the threshold described above.

Finding that the L101W and L104R H2A.Z mutations were predicted to produce long-range allosteric effects prompted us to examine if other mutations show similar effects. Expanding the AlloSigMA 2 analysis to all H2A.Z mutations that affect amino acids identical between yeast and humans revealed that only the S103Y, G113C, and H117R mutations were predicted to produce strong allosteric effects (Supplementary Figure S9A). Of these, the S103Y mutation showed allosteric effects that mirrored the effects of the L101W and L104R mutations, effects consistent with these mutations affecting amino acids close in space (Supplementary Figure S9B). Although it is surprising that the S103Y mutation did not show growth defects when introduced into yeast cells, we note that the resulting physicochemical change is relatively mild when compared to the L101W and L104R substitutions.

### H2A.Z mutations differentially impacted nucleosome structure

To validate the AlloSigMA results and understand the mechanism by which structurally distinct perturbations to H2A.Z produce graded effects on nucleosome occupancy and function at the atomistic level, we performed all-atom molecular dynamics (MD) simulations in wild-type yeast nucleosomes and nucleosomes with H2A.Z mutations. To recapitulate the native context, the DNA sequence from the X-ray structure used to generate the models was replaced with the *S. cerevisiae GIT1* promoter sequence. Three mutations were selected for this analysis: the F32Y mutation was chosen as a negative control, a largely conservative substitution that did not show growth or allosteric effects; the R36H mutation which showed defects *in vivo* but was not predicted to produce long-range allosteric effects *in silico*; and the L104R mutation which showed both growth and long-range allosteric effects.

**Figure 5:**
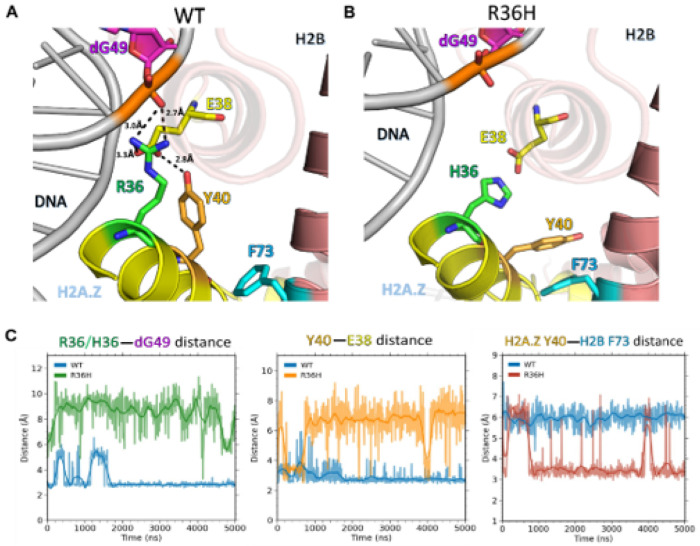
Atom-level mechanisms by which the R36H mutation influenced nucleosome structure and dynamics **(A**) Representative conformation in wild-type nucleosomes showing the H2A.Z R36 residue forming a salt bridge with DNA. (**B)** Representative conformation of the R36H mutant showing loss of the H2A.Z-DNA interaction. **(C)** Comparison of interaction distances affected by the R36H mutation. The distances are shown between H2A.Z R36 (H36 in the mutant) and DNA dG49 (blue vs. green; left), H2A.Z Y40 and H2B E38 (blue vs. orange; middle), and H2A.Z Y40 and H2B F73 (blue vs. red; right) in wild-type and the R36H mutant nucleosome.

In the wild-type nucleosome, the long and positively charged side chain of R36 on H2A.Z formed a salt bridge with the negatively charged phosphate group of nucleotide dG49 on the DNA (Fig. 5A). The R36 residue also interacted with H2B E38, a nearby negatively charged amino acid near the H2B N-terminal tail. These interactions stabilized both the H2A.Z-DNA contacts and the orientation of H2B E38, allowing H2A.Z Y40 to form stable hydrogen bonds with H2B E38. The R36H substitution abolished the interaction with the dG49 phosphate and disrupted the salt-bridge network involving dG49 on DNA, H2A.Z R36, and H2B E38(Fig. 5B). This disruption increased the distance between the H2A.Z α1 helix and DNA, weakened local interactions, and enhanced E38 side-chain dynamics, preventing stable hydrogen bonding with H2A.Z Y40 (Fig. 5C). Interestingly, in the R36H mutant, the H2A.Z Y40 residue reoriented to form a π-π stacking interaction with H2B F73 (Fig. 5B; Supplementary Figure S10A). To quantify how the R36H mutation affected histone-DNA interactions within nucleosomes, we calculated binding free energy between DNA and either the H2A.Z/H2B dimer or the complete histone octamer using the MM/GBSA method. These calculations showed that the R36H mutation weakened both H2A.Z/H2B-DNA and octamer-DNA binding compared to the wild type (Supplementary Figure S10A). Interestingly, the R36H mutation also enhanced the binding between the H3/H4 tetramer and DNA (Supplementary Figure S10A).

Focusing on the H2A.Z L104R mutation, we note that in the wild-type system, the L104 residue engaged with H2B residues F68, I104, L105, and L109 (Fig. 6A), which formed a hydrophobic pocket that accommodates the H2A.Z L104 residue and maintains interactions between H2A.Z and H2B. Upon mutation to arginine, the positively charged arginine side chain was disfavored within the H2B hydrophobic pocket and reoriented toward the solvent, forming a salt bridge with H4 D68 (Fig. 6B). This conformational rearrangement distorted the interface between the H2A.Z αC helix and the C-terminal loop that contacts the H2B α3 and αC helices. To quantify the extent of the conformational change, we calculated the dihedral angle as defined by D99 and L104 on H2A.Z and I104 on H2B. We found that in the L104R mutant, the dihedral angle changed by as much as ~ 70° (Fig. 6C). This, in turn, altered the conformational orientation of the αC and part of the α3 helix of H2A.Z which includes the M6 region important for SWR1 binding. Importantly, this region of H2A.Z exhibited significantly increased Root Mean Square Deviation (RMSD) values in the L104R mutant compared to the wild type (Fig. 6D). Significant increases in RMSD values were also observed in the α2 helix of the protein although the magnitude of these effects was weaker compared to that observed in the αC helix.

As several acidic patch residues are located within the regions showing conformational changes, this raised the possibility that solvent-accessible surface area (SASA) would be affected. Consistently, SASA values for the acidic patch residues E98 and E100 increased from a mean of 99.5 Å^2^ in wild type to 139.2 Å^2^ in the L104R mutant (Fig. 6E). Given that acidic patch residues function as key docking sites for chromatin-associated factors[73], our findings suggest that the L104R mutation may perturb nucleosome-binding factor interactions. Given that the L104R mutation distorted the H2A.Z αC helix and rearranged the H2A.Z-H2B interface, we again wondered if this ultimately influenced histone-DNA interactions. As before, binding free energy between DNA and either the H2A.Z/H2B dimer or the complete histone octamer was calculated. The MM/GBSA calculations showed that the L104R mutation weakened H2A.Z/H2B-DNA and octamer-DNA binding (the absolute binding energy value was lower) compared to the wild type (Supplementary Figure S10B). Again, it was interesting that the binding between the H3/H4 tetramer and DNA was increased in the mutant compared to the wild type (Supplementary Figure S10B). In contrast to the effects observed for the R36H and L104R mutations, and indicative of not all cancer mutations producing effects on nucleosome structure, the F32Y mutation did not show noticeable conformational changes. Consistently, histone-DNA binding free energy showed no appreciable differences when wild-type and F32Y nucleosomes were compared (Supplementary Figure S10C).

### H2A.Z mutations disrupted nucleosome stability

**Figure 7:**
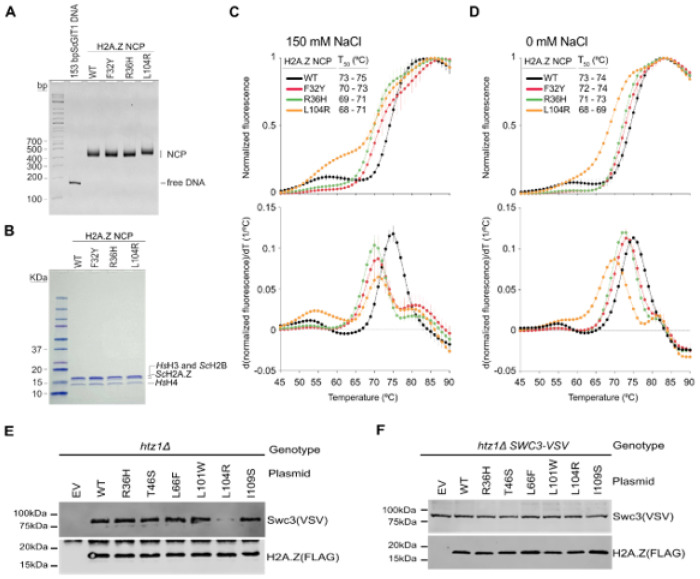
H2A.Z mutations reduced nucleosome stability and impaired SWR1-C interaction **(A**) 5% native PAGE stained with ethidium bromide showing the indicated H2A.Z nucleosome core particles (NCP) reconstituted by salt dialysis *in vitro*. The gel shows a single nucleosome species for each of the H2A.Z variants that migrates slower in the gel than the protein-free *Sc*GIT1 DNA. (**B**) 4-20% SDS-PAGE stained with Coomassie blue of the indicated H2A.Z NCPs samples as in **A**. *H. sapiens* (*Hs*) histone H3 and H4, and *S. cerevisiae* (*Sc*) histone H2B and H2A.Z polypeptides were used to reconstitute nucleosomes. The gel shows the expected molecular weight for the histone proteins indicating no protein degradation after NCP reconstitution. **(C-D)** Thermal stability shift assays of H2A.Z nucleosomes in 150 mM NaCl (**C**) and 0 mM NaCl (**D**). The top graphs show the thermal denaturation profile of nucleosome samples by plotting the normalized SYPRO orange signal as a function of temperature. T_50_ corresponds to the temperature at which the normalized fluorescence signal is 0.5 (50%). The bottom plots show the differential values of the thermal stability curves presented at the top to reveal the inflection points in the thermal stability curve, which correspond to the nucleosome transition unfolding steps. Data from at least three experiments were used to calculate the mean and standard deviation (error bars). All H2A.Z mutations generally shifted the thermal denaturation profile of the NCP towards lower temperatures when compared to wild type, especially the main denaturation peak around 70 – 75 °C indicating that these mutations generally decrease the stability of the nucleosome. **(E**) Immunoprecipitation of FLAG-tagged wild-type or mutant H2A.Z revealed defects in SWR1-C binding for the L104R mutant. Swc3 was VSV tagged and used as a representative subunit SWR1-C. **(F**) Representative immunoblots of whole-cell extracts generated from *htz1Δ* SWC3-VSV strains carrying the indicated plasmids show normal levels of H2A.Z and Swc3 in wild type and the H2A.Z mutants. The *htz1Δ* strain carrying an EV served as a negative control.

Finding that the R36H and L104R mutations weakened H2A.Z/H2B-DNA and octamer-DNA binding *in silico* prompted us to examine their effects on nucleosome stability *in vitro*. Nucleosome core particles (NCPs) were reconstituted using purified components and wild-type or R36H, L104R, or F32Y mutant H2A.Z. To mimic the native environment, 147 bp of *Sc*GIT1 DNA sequence was generated and used for H2A.Z nucleosome reconstitution *in vitro* (Fig. 7A). Initial nucleosome reconstitution by salt dialysis using the 153 bp *ScGIT1* DNA and only *S. cerevisiae* histone proteins yielded heterogeneous histone-DNA products by native gel electrophoresis. Although the specific histone composition could not be accurately assigned (data not shown), these were likely subnucleosomal particles and aggregates resulting from suboptimal nucleosome reconstitution conditions. However, we note that *in vitro* yeast nucleosome reconstitution is known to be challenging due to the highly dynamic and unstable H3/H4 tetramer structure[74]. To overcome this challenge, *Hs*H3, *Hs*H4, *Sc*H2A, *Sc*H2B, and the 153 bp *Sc*GIT1 DNA we used, resulting in unambiguous nucleosomes by salt dialysis where H2A.Z was either wild type or F32Y, R36H, or L104R mutants (Fig. 7A and 7B).

To determine whether the amino acid substitutions affected overall nucleosome stability, we used thermal stability shift assays[75]. Regardless of the mutation, NCPs harboring H2A.Z amino acid substitutions exhibited a different denaturation profile at both 150 and 0 mM NaCl when compared to the wild type with a general shift towards lower temperatures (Fig. 7C and 7D). In addition, while the H2A.Z wild-type NCP showed a biphasic denaturation profile with differential peaks at about 52–55 °C and 75 °C, the H2A.Z mutant NCPs had an additional differential peak at about 82 °C (Fig. 7C and 7D), indicating that the F32Y, R36H, and L104R H2A.Z mutations change the pathway of nucleosome denaturation under increasing temperature. The appearance of multiple phases in the thermal denaturation profile of nucleosome samples has been interpreted as the uncoupled and independent denaturation of histone modules within the nucleosome, such as the H2A/H2B dimer and the H3/H4 tetramer and can vary between nucleosome variants[36, 75].

Given that differences in denaturation pathways do not necessarily relate to overall stability, we determined the temperature at which 50% of the nucleosome sample was denatured (T_50_) (i.e., the temperature at which the normalized fluorescence is 0.5), as a proxy for global thermal stability of the nucleosomes (Fig. 7C and 7D). At 150 mM NaCl, the wild-type H2A.Z nucleosome had a T_50_ range of 73–75 °C. In contrast, the T_50_ decreased by 2–3 °C for F32Y, 4 °C for R36H, and 4–5 °C for L104R nucleosomes when compared to the wild type (Fig. 7C), indicating that the mutations decrease the thermal stability of the NCP, with F32Y being less severe. The same trend between the wild-type and H2A.Z mutants was observed for T_50_ values at 0 mM NaCl conditions, except that F32Y and R36H behaved more similarly to the wild type, showing only 1 °C and 1–2 °C difference between their T_50_ values compared to the wild type, respectively (Fig. 7D). Collectively, these results showed a clear hierarchy of nucleosome destabilization, with minimal effects seen for the F32Y mutation and more pronounced effects for R36H and L104R, effects consistent with MD-predicted structural perturbations and the observations *in vivo*.

### The L104R mutation decreased interaction with the SWR1 complex

Finding that the L104R mutation disrupted the conformation of the M6 region critical for SWR1 binding *in silico*[25, 28, 29] prompted us to examine if it disrupts binding *in vivo*. To this end, plasmids carrying wild-type or mutant *HTZ1* were introduced into a *htz1Δ SWC3-VSV* strain. An empty vector control was used as a negative control. FLAG-tagged H2A.Z was purified and the levels of co-purifying Swc3, a subunit of the SWR1 complex, were determined via immunoblotting (Fig. 7E). Consistent with previous reports, wild-type H2A.Z is associated with Swc3[28, 48, 54]. Importantly, no Swc3 co-purified with H2A.Z in strains lacking *HTZ1-FLAG*. Most of the mutants showed levels of co-purifying Swc3 similar to wild type except for the L104R mutant which decreased but did not completely abolish Swc3 interactions (Fig. 7E). Importantly, the decrease in the amount of Swc3 captured by H2A.Z in the L104R mutant was not simply a result of changes to total levels of Swc3 protein, as these were comparable to wild type in whole cell extracts (Fig. 7F). These findings validate the MD simulation results and together with the *in vitro* stability assays, indicate that genome-wide H2A.Z occupancy defects in this mutant likely arise from combined defects in nucleosome deposition and stability. Finding that all other H2A.Z mutants captured normal levels of the SWR1 subunit suggests that their changes in genome-wide occupancy may reflect defects in nucleosome stability rather than deposition.

#### The effects of the R36H and L104R mutants on chromatin association were conserved in human cells

**Figure 8:**
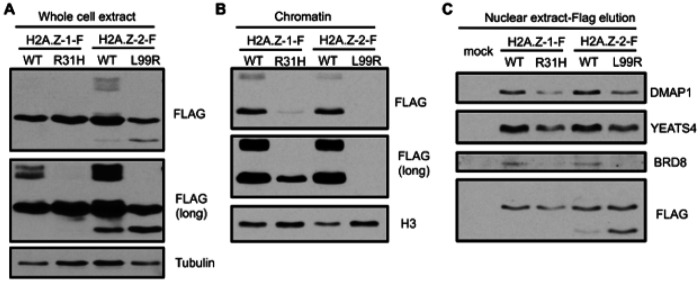
H2A.Z mutations have similar effects in human cells Isogenic human K562 cell lines expressing FLAG-tagged wild-type and mutant H2A.Z-1/2 from the AAVS1 safe harbor locus were fractionated and analyzed by immunoblotting with the indicated antibodies. **(A)** Whole cell extracts (6 μg). **(B)** Chromatin extracts (0.5 μg). **(C)** Elution of an anti-FLAG immunoprecipitation from a soluble nuclear extract. Mock shows the results from a cell line expressing an empty FLAG vector. DMAP1 and YEATS4 are common subunits of SRCAP and EP400 complexes, while BRD8 is a subunit representative of the EP400 complex.

To determine if the effects of the H2A.Z mutations seen in yeast were conserved in human cells we used genome editing to produce isogenic cell lines expressing wild-type and mutant human H2A.Z paralogs from the *AAVS1* safe harbour in the K562 cellular background. H2A.Z-1 and H2A.Z-2 were tagged at their C-termini with a triple-FLAG epitope, as previously described[76]. The H2A.Z-1 R31H (R36H in yeast) and H2A.Z-2 L99R (L104R in yeast) mutations were compared to their respective wild-types. Interestingly, ubiquitinated H2A.Z-1/2 was detected in the whole cell extracts of wild-type proteins but not the mutants (Fig. 8A). Furthermore, a short isoform of H2A.Z-2 was increased in the presence of the L99R mutation. Chromatin extracts were prepared with MNase and soluble proteins were removed to visualize protein levels in chromatin. Both mutants had a clear decrease in chromatin association compared to their respective wild types (Fig. 8B). Although some H2A.Z-1 R31H was detected in chromatin, none was seen with the H2A.Z-2 L99R mutant, results consistent with findings in the yeast model system. We then investigated if these mutants affected the interaction with the protein complexes functionally homologous to yeast SWR1. We previously showed that soluble human endogenous H2A.Z-1/2-H2B dimers in nuclear extracts are associated with the SRCAP and EP400/TIP60 ATP-dependent chromatin remodelling complexes, the latter showing preference for H2A.Z-2[77]. Thus, we prepared nuclear extracts from the cell lines above and performed anti-FLAG immunoprecipitations followed by native elution with FLAG peptides and analysis by immunoblotting. As shown in Fig. 8C, common subunits of SRCAP and EP400 (DMAP1 and YEATS4, homologous to Swc4 and Yaf9 in yeast SWR1) co-purified with wild-type H2A.Z-1 and − 2. In contrast, the L99R mutant showed decreased association with SRCAP and EP400 mirroring the effects seen in yeast. Although a slight decrease in association with SRCAP and EP400 subunits was detected in the H2A.Z-1 R31H mutant, we note that the levels of H2A.Z captured in that mutant were lower than wild type and thus the effects in part reflect differences in starting material. Altogether, these results indicate that the functional analysis of cancer-associated H2A.Z mutations in the yeast model system is directly relevant to understanding their impact in humans.

## Discussion

H2A.Z is a histone variant with central roles in shaping chromatin structure and function, but whose exact mode of action remains incompletely understood. Here, we used cancer missense mutations to probe H2A.Z structure-function relationships while also uncovering mechanisms by which these alterations may contribute to oncogenesis. Systematic analysis of cancer-associated H2A.Z mutations in the budding yeast model system revealed alterations at six amino acids that disrupted normal function. Importantly, most of these amino acids were not known to contribute to H2A.Z activity. Follow-up analysis showed that these mutations decreased genome-wide occupancy in part by disrupting nucleosome dynamics, stability, and interaction with nucleosome-binding proteins, the latter two recapitulated in human cell lines. Collectively, this work identified new amino acids important for normal H2A.Z function, highlighting the power of the cancer missense mutations in revealing novel insight about histone biology. This work further underscores the utility of yeast as a model organism for understanding the impact of cancer histone mutations[38, 78, 79] by showing that findings captured in this system recapitulate effects in humans where these alterations may contribute to disease.

Our results are consistent with previous reports highlighting I109 as a critical amino acid for normal H2A.Z function. We found that the I109S mutation recapitulated the growth defects seen for the I109A (from an alanine scan) and I109T (from a random mutagenesis screen) substitutions upon exposure to HU, MMS, benomyl, and caffeine[46, 47]. The I109S mutant also recapitulated the growth defects seen in formamide and the bulk chromatin occupancy defects of the I109T, phenotypes not tested for the I109A mutant. Our work extends these findings by showing that the I109S mutation also produced growth defects upon exposure to cold and osmotic stress, suggesting that the same phenotypes may be seen for the I109A/T mutants if tested. The phenotypic similarity of the I109A/T/S mutations likely reflects the similar physicochemical properties of the substituting amino acids. Nevertheless, we do note one key difference, while previous work showed that the I109T mutation decreased binding to SWR1 complex subunits, the same effect was not seen with the I109S cancer mutation[46]. While it remains unclear why this may be the case, these results are in line with growing evidence highlighting the importance of the mutant amino acid in shaping phenotypic outcomes[79–81]. Consistently, we found that the R36H, T46S, L66F, L101W, and L104R mutations produced growth defects despite previous reports that alanine substitutions at the same amino acid positions show no growth defects[47]. The latter highlights the power of the vast cancer mutational landscape to complement previous structure-function efforts.

Importantly, the mutants did not phenocopy the complete loss of H2A.Z (the *htz1Δ* mutant) or the inability to deposit H2A.Z into chromatin (the *swr1Δ* mutant) suggesting that the effects were not merely a result of a lack of H2A in chromatin. In fact, removing *SWR1* in strains carrying H2A.Z mutations abolished their growth effects, indicating that their effects emerged from their presence on nucleosomes. Finding that the mutants also produced different effects on growth and chromatin occupancy, it suggested that they disrupted function to different extents or via distinct mechanisms. In support of the latter, high-resolution all-atom dynamic simulations showed that the R36H and L104R mutants produced distinct effects on H2A.Z-containing nucleosome structure and dynamics. Specifically, R36H disrupted histone-DNA contacts while L104R produced large conformational rearrangements. Although both mutants ultimately disrupted nucleosome stability *in vitro* they did so to different extents that were mirrored *in vivo*.

Beyond affecting stability, the L104R mutation was also predicted to produce large-scale conformational rearrangements that altered the position and surface accessibility of acidic patch residues important for interaction with nucleosome-binding proteins. Validating the ability of histone mutations to disrupt function through long-range allosteric effects, we showed that this mutant decreased the ability to interact with SWR1 complex subunits *in vivo* in both yeast and human cells. These findings are in line with SWR1 requiring a region of H2A.Z that includes several acidic patch residues for physical interaction[25, 46, 48]. Finding that the other mutants showed SWR1 binding and genome-wide deposition patterns similar to wild type suggests that chromatin incorporation may be largely unaffected by the mutations. Instead, the observed changes in chromatin occupancy may reflect different extents of decreased nucleosome stability.

Although the high computational demands of the all-atom simulations prevented us from acquiring atom-level insight about the effect of the mutations on nucleosome structure and dynamics, the network and allosteric-effect analysis provided important insight that supported the idea that each mutation disrupts function through a unique mechanism. Finding that mutations on T46, L66, and I109 were not predicted to produce long-range allosteric effects indicated that their effects on function most likely emerge from alterations to immediate contacts. For mutations on T46 (equivalent to human T41), an amino acid on the L1 loop which sits at the H2A-H2B dimer interface on the nucleosome[8, 11], this may include weakening dimer stability. For mutations on I109 (equivalent to I105 in humans), this may involve altering interactions with ANP32E, a histone chaperone critical for H2A.Z deposition into chromatin[64, 65, 82], as well as altering interactions with H3 and H4 in the context of the nucleosome. For mutations on L101 (equivalent to human L96), an amino acid that makes contacts with the KMT5B H4K20 methyltransferase[63], this may include defects on this PTM which plays important roles in transcription regulation, establishing constitutive heterochromatin at pericentric regions, and maintaining genome integrity[83–86]. Beyond the predicted effects of L101 on PTMs, our results show that the R31H and L99R mutations decreased H2A.Z ubiquitination, a PTM involved in transcription regulation and DNA damage repair[87–89] that is deposited by RING1B, a protein that binds nucleosomes via direct contacts with several acidic patch residues[89, 90]. Thus, our work adds to the growing body of evidence showing that a common theme by which cancer histone mutations disrupt function is by disrupting PMTs[35, 71, 91, 92].

Although our analysis of cancer histone mutations identified novel residues critical for H2A.Z function, several cancer mutations affecting amino acids previously shown to be important for H2A.Z activity were surprisingly well tolerated: F32Y, L81V/I, R85C, and G113C[36, 47]. The F32Y mutant showed no growth defects despite mild nucleosome destabilization *in vitro* and prior alanine substitutions showing sensitivity to MMS and caffeine. The L81I/V and G113C mutants had no clear effects under the conditions tested, yet their equivalent alanine substitutions showed sensitivity to MMS. Similarly, the R85C mutant (human R80C) showed no growth defects under the conditions tested, despite previous evidence of destabilizing the nucleosomes *in vitro* and our work showing that it reduced chromatin association *in vivo*. The discrepancies observed for F32, L81, and G113 mutations may reflect differences in the substituting amino acid as all previous insight was derived from alanine substitutions. For the R85C mutation (R80C in humans), the mild impact on nucleosome stability compared to the L66F, L101W, L104R, and I109S mutants may be insufficient to drive growth defects *in vivo*. While these findings highlight the power of bulk growth assays to identify mutations causing strong functional defects, it may overlook mutations that produce more subtle effects. As such, further insights into H2A.Z structure and function may be gained by testing the remaining mutants using complementary assays, including methods to measure chromatin association and nucleosome stability, defects that emerging as common mechanisms by which cancer-associated H2A.Z mutations disrupt function.

Although our study focused on H2A.Z, the functional effects observed may extend to its replication-dependent cousin given that the residues we identified as critical for H2A.Z function are conserved in H2A in both yeast and humans. In yeast, H2A.Z R36 corresponds to H2A R30; L66 to L59; L101 to L94; L104 to L97; and I109 to I103. The only mutated residue not conserved between yeast and humans is T46 (T41 in humans) in H2A.Z which corresponds to N39 (N40 in humans) in H2A. Although previous alanine scans of H2A and H2A.Z showed that mutating R36, L66, and L101 in H2A.Z caused no detectable phenotypes, the equivalent substitutions in H2A (R30, L59, and L94) led to growth defects under a wide range of conditions[47, 93–95], revealing that alterations in H2A have more severe effects than the equivalent substitutions in H2A.Z. Nevertheless, we note that despite being conserved, the H2A L97A mutation which affects the H2A.Z L104 equivalent position did not produce growth defects, findings that may reflect the fact that L104 resides in the M6 segment, a region that confers H2A.Z-specific functions[25, 28, 48, 96, 97].

By characterizing the functional and structural impact of cancer histone H2A.Z mutations, our work contributes to our understanding of the impact of cancer histone mutations. Here we report cancer histone mutations on H2A.Z that disrupt PTMs, interactions with chromatin remodelers, and nucleosome stability findings consistent with emerging themes in the field[7, 12, 15, 32, 35, 36, 38, 42, 66, 71, 79, 91].

## Materials and Methods

### Yeast and human H2A.Z protein sequence alignments

Multiple sequence alignments from HistoneDB[98] were used to identify amino acid positions in the *S. cerevisiae* histone H2A.Z protein sequence that correspond to residue mutations in the human version of the protein.

### Yeast strains and plasmids

The wild type, *htz1Δ*, and *htz1Δ SWC3-VSV* W303 strains were described previously[25, 48]. All plasmids used in this study are listed in Supplementary Table S6 and were introduced into yeast cells by transformation[99]. Centromeric low-copy pRS314 expression vectors[100] carrying wild-type or mutant *HTZ1* driven by 414 bp of endogenous *HTZ1* promoter sequence and fused in-frame to a 3xFLAG sequence were generated by recombining PCR fragments into the linearized empty vector via homologous recombination using a modified version of the method described by van Leeuwen et al[101]. All primers used in this study are listed in Supplementary Table S7. All strains used in this study are listed in Supplementary Table S8.

### Yeast growth assay

Overnight cultures were grown in SC-TRP liquid medium. The next morning, they were diluted to 0.5 OD_600_, 10-fold serially diluted, and spotted onto CS-TRP solid media with and without the indicated amounts of benomyl (Cat. 45339, Sigma), caffeine (Cat. C0750, Sigma), formamide (Cat. 47671, Sigma), HU (Cat. HB0528, Bio Basic), MMS (Cat. 129925, Sigma), or NaCl (Cat. SOD001, BioShop). Plates were incubated at 30 °C or the indicated temperatures for 2–4 days and imaged with a flatbed scanner.

### Yeast whole cell extracts and immunoblotting

Whole-cell extracts were prepared by glass bead lysis in the presence of trichloroacetic acid (Cat. TCA001, BioShop) as reported previously[102]. Immunoblotting was performed using the anti-FLAG M2 (Cat. F3165, Sigma) antibody. The Revert 700 Total Protein Stain (Cat. 926-11016, LICOR) was used to assess protein loading. Membranes were imaged using a LICOR XF scanner.

### Chromatin association assay

Chromatin association assays were performed as described previously[103]. Overnight cultures were diluted to an OD_600_ of 0.15 in 50 mL of SC-TRP medium and grown to an OD_600_ of ~ 0.5. A total of 20 OD units were harvested, incubated in pre-spheroplast buffer (100 mM PIPES-KOH pH 9.4, 10 mM DTT, and 0.1% sodium azide) for 10 min at room temperature, and spheroplasted with 200 mg Zymolyase-100T (Cat.120493-1, Amsbio) in spheroplast buffer (50 mM KPO_4_ pH 7.5, 0.6 M sorbitol, and 10 mM DTT) for 30 min at 37 °C with rotation. Spheroplasts were washed with wash buffer (50 mM HEPES-KOH pH 7.5, 0.4 M sorbitol, 2.5 mM MgCl_2_, and 100 mM KCl), resuspended in EB (50 mM HEPES-KOH pH 7.5, 2.5 mM MgCl_2_, 100 mM KCl, 1 mM DTT, 1 mM PMSF, 2 μg/mL aprotinin, and 1 μg/mL leupeptin), and lysed on ice with 0.1% Triton X-100. A portion of the whole cell extract (WCE) was saved, and the remainder was centrifuged through EBSX (EB + 0.25% Triton X-100 and 30% sucrose) to separate chromatin and supernatant fractions. The WCE, chromatin, and supernatant fractions were analyzed by SDS-PAGE and immunoblotted with anti-Pgk1 (Cat. Ab113687, Abcam), anti-FLAG (Cat. PA1-984B, ThermoFisher SCITIFIC), and anti-H4 (Cat. Ab31830, Abcam) antibodies.

### Co-immunoprecipitation

Overnight cultures were grown at 30 ℃, diluted to an OD_600_ of 0.05 in 100 mL of SC-TRP, and grown to an OD_600_ of 0.8–1.2. One hundred OD_600_ units were collected by centrifugation per sample, and the cell pellet was washed with 10 mL of ice-cold PBS pH 7.4 (137 mM NaCl, 2.7 mM KCl, 10 mM Na_2_HPO_4_, and 1.8 mM KH_2_PO_4_). The cell pellet was resuspended in 0.6 mL of ice-cold TAP-IP buffer (50 mM Tris, 0.5 M NaCl, 1.5 mM MgAc, 10 mM NaPPi, 5 mM EDTA, 5 mM EGTA, 5 mM NaF, 0.1 mM Sodium orthovanadate, 10% glycerol, 0.15% NP-40, 0.2 mM DTT, and protease inhibitor), and approximately 0.75 mL of acid-washed glass beads were added. Cell lysis was performed by bead beating 5 times at 4 °C for 1 min, letting the sample rest for 1 min between cycles (Cat. 607, BIOSPEC PRODUCTS). The supernatant was transferred to a new microcentrifuge tube and centrifuged at 14,000 rpm for 20 min at 4 °C. The supernatant was added to the prepared FLAG IgG beads and incubated for 2 hr at 4 °C with gentle rotation. The beads were washed 3x with 1 mL of cold TAP-IP buffer, resuspended in 50 μL of 3× SDS sample buffer, boiled for 5 min at 95 °C, and centrifuged at full speed for 5 min. The supernatant was loaded onto an SDS-PAGE gel and blotted with anti-FLAG M2 (Cat. F3165, Sigma) and anti-V5 (Cat. MA5-15253, Invitrogen) antibodies to detect H2A.Z and Swc3, respectively.

### Chromatin immunoprecipitation (ChIP) and quantitative PCR (qPCR)

ChIP experiments were performed as described previously[104]. Briefly, overnight cultures were diluted to 0.15 OD_600_ in 250 mL of SC-TRP media and grown to an OD_600_ of ~ 0.7. Cells were cross-linked with 1% formaldehyde for 20 min, and cross-linking was quenched by adding glycine to a final concentration of 300 mM and incubating for 5 min. Cells were washed twice with cold Tris-buffered saline (20 mM Tris-HCl pH 7.5 and 150 mM NaCl) and once with ice-cold lysis FA lysis buffer (50 mM HEPES-KOH pH 7.5, 140 mM NaCl, 1 mM EDTA pH 8.0, 1% Triton X-100, 0.1% Na-deoxycholate, 0.1% SDS, and protease inhibitors). Cell pellets were resuspended in 800 μL of ice-cold FA lysis buffer and *S. pombe* cells expressing FLAG-tagged Rpb3[105] were spiked into each sample at a 1:10 ratio. Cells were disrupted by bead-beating (Cat. 607, BIOSPEC PRODUCTS). Chromatin was sonicated (10 cycles of 30 s on/off, high setting) using a Bioruptor Pico sonication device (Cat. B01080010, Diagenode) and incubated with 3 μL of anti-FLAG M2 antibody (Cat. F3165, Sigma) coupled to 30 μL of Protein A Dynabeads (Cat. 10002D, Invitrogen) for 3 hr at 4°C with gentle rotation. Beads were washed three times with cold FA lysis buffer, three times with cold FA lysis buffer with 360 mM NaCl, and once with cold Tris-EDTA (10 mM Tris-HCl pH 8.0 and 1 mM EDTA pH 8.0). Chromatin was eluted using elution buffer (50 mM Tris-HCl pH 8.0, 10 mM EDTA, and 1% SDS) and incubated at 65°C for 15 min. Reverse cross-linking was performed by incubating eluted chromatin with 3 μL of RNase Cocktail Enzyme Mix (Cat. AM2288, Invitrogen) and 1% SDS at 65°C overnight. Proteinase K treatment was performed for 1.5 hr at 42°C. DNA was recovered using the QIAGEN PCR purification kit (Cat. 28104, QIAGEN).

Biological triplicates were performed for all ChIP-qPCR and ChIP-seq experiments. All samples were normalized to the spike-in control. Primers used for qPCR are listed in Supplementary Table S7. Both the input and immunoprecipitated materials were sequenced for ChIP-seq. ChIP library construction and sequencing (paired-end 150 bp, Illumina NovaSeq) were performed at the Canada’s Michael Smith Genome Sciences Centre, Vancouver, Canada.

### ChIP-seq data analysis

Raw and normalized ChIP-seq data can be accessed at the European Nucleotide Archive (ENA) at MBL-EBI under accession number ERP183505. ChIP-seq datasets were analyzed as described previously[20, 106] with the following modifications. Briefly, fastp (0.23.4)[107] was used to confirm the quality of the raw reads. Reads were aligned to a merged genome made up of the *S. cerevisiae* (SaCcer3 R64-5-1) and *S. pombe* reference genomes (ASM294v3) using Bowtie2[108] with default settings. PCR duplicates were removed using samtools[109]. Total read counts of each sample are listed in Supplementary Table S3. Normalization factors were computed based on the proportion of reads mapped to the *S. pombe* genome as described previously[110]. The multiBigwigSummary (bin size = 100) followed by plotCorrelation functions from the deepTools suite (3.5.6)[111] was used to assess reproducibility across biological replicates. The bigWig files were generated using the bamCoverage function and used to visualize the ChIP signal across the genome. The three biological replicates for each sample were merged into one bigWig using bigwigAverage. Enrichment tracks were visualized using Integrated Genome Browser (IGB; 10.1.0)[112]. Heatmaps and binding profiles were generated using deepTools[111] and the R64-5-1 *S. cerevisiae* gene annotation[113].

Peaks were called on the individual biological replicate using MACS3 (3.0.3)[114] with the following parameters: -t [treatment file] -c [control files] -g 1.2e7 -bdg -q 0.05 -f BAMPE. Both the sample input and empty vector immunoprecipitation files were used as controls. Reproducible peaks across replicates were identified using irreproducible discovery rate (IDR) analysis (2.0.4.2)[115]. Pairwise comparisons between replicates were performed using -rank q.value and -idr-threshold 0.05. Peaks passing the IDR threshold of 0.05 were considered reproducible. The final list of reproducible peaks was obtained by intersecting the pairwise IDR peak sets using BEDTools (2.31.1)[116] with default settings and visualized as an UpSet plot using the intervene UpSet module (0.6.5). The nearest transcription start site to each peak summit was identified using annotatePeaks.pl with a parameter -size 0 in HOMER (5.1)[117]. The results are provided in Supplementary Table S5.

### RT-qPCR

Overnight yeast cultures were diluted to 0.15 OD_600_ in 25 mL of SC-TRP. Cells were grown to an OD_600_ of ~ 0.5, and a total of 10 OD_600_ units were collected. RNA was extracted using the RNeasy Mini Kit (Cat. 74104, Qiagen) and converted to cDNA using the QuantiTect Reverse Transcription Kit (Cat. 205311, Qiagen). mRNA levels were quantified with SsoAdvanced Universal SYBR Green Supermix (Cat. 1725270, BIO-RAD) according to the manufacturer’s protocol. Samples were analyzed from three independent biological replicates and normalized to *ACT1*. Primer sequences are listed in Supplementary Table S7.

### Construction of H2A.Z interaction subgraphs

A histone-centric interactome was reconstructed from experimentally determined complexes in PDB retrieved in January 2025, following the Histone Structural Interactome pipeline[10] with minor adaptations. First, we queried the PDB for all structures containing canonical or variant histones and downloaded the corresponding biological assemblies. We retained core-layer human histone complexes and their primary and secondary binding partners, while excluding stabilizing fragments (e.g., scFv), immune-related components, viral proteins, synthetic or pharmaceutical peptides, proteins lacking UniProt annotation, mutant histones, MHC/HLA-associated histones, and ambiguous peptides. Only chains that could be reliably aligned to canonical UniProt sequences were retained. Next, for each retained biological assembly, we identified physical contacts by computing all heavy-atom distances between residue pairs across different chains or within the same chain but separated by more than one position along the sequence. Physical contacts were retained if the distances were ≤ 5 Å and residue pairs exhibiting steric clashes or geometric artifacts were removed. All contacts observed across structures were merged into a unified residue-residue interaction network, in which nodes represent individual amino acids and edges represent experimentally observed physical contacts. For examining H2A.Z, we extracted the subnetwork of contacts directly involving H2A.Z residues and restricted all proteins to human entries. The PDB structures included in the H2A.Z analysis were: 1F66, 3WA9, 3WAA, 4CAY, 5B31, 5B32, 5B33, 5CHL, 5FUG, 6M4D, 7YRD, 7YRG, 8JHF, 8T9F, and 8THU.

### AlloSigMA analysis

All available homotypic H2A.Z nucleosome structures (PDBs: 5B33, 3WA9, 3WAA, and 1F66) and heterotypic H2A/H2A.Z structures (PDBs: 5B31 and 5B32) were used in the AlloSigMA 2 server to compute residue-level allosteric free energy changes (Δh)[72]. H2A.Z mutations were modelled as UP (an increase in side-chain volume) or DOWN-mutations (a decrease in side-chain volume). The K3N mutation was excluded because disordered tail regions are absent in all available structures. The L81I equivalent mutation was also excluded because leucine and isoleucine are structural isomers, making the mutation unsuitable for meaningful UP/DOWN analysis.

To account for minor variations in sequence lengths across PDB files, residues were aligned to a reference structure to ensure all sequences are the same length. For the homotypic set, PDB 5B33 was used as the reference; for the heterotypic set, PDB 5B31 was used as the reference since these structures contain the fewest amino acids. Residues missing in the reference structure but present in the non-reference structures were excluded from the analysis. Residues present in the reference but absent in the non-reference structures were assigned an allosteric free energy value of 0. Per-residue Δh values across the two heterotypic structures or four homotypic structures with one or two mutations were averaged, and the standard deviation was computed. Averaged Δh values were mapped onto the reference structures using a custom Python script that converts Δh values to an RGB format compatible with PyMOL. The results were visualized in PyMOL (v3.1.0)[118]. Average allosteric effects were plotted using the ggplot2 package (v3.5.2)[119] in R (v.4.5.0)[120].

### Wild-type and mutant nucleosome modelling

Wild-type and mutant nucleosome models were generated as follows. Because there are no available experimental structures of a yeast nucleosome containing H2A.Z, an X-ray crystal structure of the yeast nucleosome core particle (PDB ID: 1ID3[121]) was used as the template. Given the high sequence similarity between yeast and human H2A.Z, a structural model of yeast H2A.Z was generated from an available structure of a human H2A.Z nucleosome (PDB ID: 1F66[122]). Both copies of canonical H2A in the 1ID3 yeast nucleosome were replaced with the yeast H2A.Z model. Histone tails were extended linearly into the solvent and symmetrically oriented with respect to the dyad axis using ChimeraX[123]. The original DNA sequence in 1ID3 was replaced with the *S. cerevisiae GIT1* promoter sequence shown below using w3DNA[124]. Finally, F32Y, R36H, and L104R mutations were introduced into both copies of H2A.Z to generate the corresponding mutant nucleosomes.

TAGTAATAGCGGCGTAGAATGTTGTACCCAACAGCAATGATGTTAGAATAATAGGGGTTATATGATTGGATCCTACGGTCTTCCGCGTTGCCGTTTGGTGCCACATTTATCTTCATATTCATGAATTTCCTTACTGGACCCCCACCT

### Molecular dynamics simulation

All MD simulations were conducted using the GPU-accelerated GROMACS version 2022.6[125]. For the protein component, we used the AMBER ff14SB force field[126]. For the double-stranded DNA, the OL15 force field was selected[127]. The Optimal Point Charge (OPC) water model was used to simulate the explicit solvent environment. For each simulation system, the initial nucleosome model was solvated within a cubic box with a minimum distance of 20 Å between any nucleosome atom and the box edges. To mimic physiological conditions the ionic strength of the system was set to 150 mM NaCl. The solvated systems were first subjected to energy minimization using 50,000 steps of steepest descent to remove any steric clashes or unfavorable contacts. Following this, a gradual heating protocol was applied, where the temperature was slowly raised to 298 K over the course of 800 ps using restraints. After heating, the systems underwent an equilibration period for 1 ns. This was followed by production simulations in the isobaric-isothermic (NPT) ensemble, maintained at a constant temperature of 298 K using the modified Berendsen thermostat[128]. The system pressure was kept constant at 1 atm through the Parrinello-Rahman barostat[129]. A cutoff distance of 10 Å was applied to manage short-range non-bonded van der Waals interactions, while the Particle Mesh Ewald (PME) method was employed to compute long-range electrostatic interactions[130]. Periodic boundary conditions were implemented. Covalent bonds involving hydrogen atoms were constrained to their equilibrium lengths using the LINCS algorithm[131]. Coordinates of the solutes were saved every 100 ps, and each simulation was performed for 5 μs, resulting in a comprehensive dataset of 50,000 frames per simulation for subsequent analysis. To ensure the robustness of our findings, three independent simulation runs were conducted for each system.

### Analysis of nucleosome dynamics

VMD scripts and Gromacs tools were used to analyze the MD trajectories[125, 132]. MD trajectory snapshots were first processed by performing a root mean square deviation fit of the C-α atoms of the histone core to the initial structure of the nucleosome. The interaction energy between the histone octamer and DNA was determined using the molecular mechanics generalized Born surface area (MM/GBSA) method[133], which is embedded in the Amber22 Package[134]. The salt concentration was assigned to 150 mM, and the generalized Born model OBC2 was used with parameters igb = 5 and radii = mbondi2. The fast LCPO algorithm was applied to compute an analytical approximation to the solvent accessible area of the molecule (molsurf = 0). The calculations were performed for each MD simulation system based on every 1 ns frame.

#### Expression and purification of recombinant histone proteins from E. coli

Plasmids for bacterial expression of *Hs*H3.2 (UniProt: Q71DI3), *Hs*H4 (UniProt: P62805), *Sc*H2B.1 (UniProt: P02293), and wild-type or mutant *Sc*H2A.Z (UniProt: Q12692) were generated using the Gateway technology subcloning system (Thermo Fisher Scientific). cDNA for each histone was manufactured by Integrated DNA Technologies (Supplementary Table S9) and subcloned into the pDONR/Zeo and pDEST17 vectors followed by Sanger sequencing for verification (Supplementary Table S6). The resulting pDEST17 expression vectors produced histone proteins with an N-terminal 6x-Histidine (His_6_) tag and a TEV cleavage site immediately upstream of the histone sequence. Removal of the tag by TEV protease cleavage resulted in a histone polypeptide with no deviations from the native protein sequence.

Expression of His_6_-tagged histone proteins was carried out in Rosetta 2 (DE3) pLysS cells cultured in 2XYT media at 37 °C. Bacterial cultures were induced with 0.5 mM IPTG when the culture reached an OD_600_ of 1.0 and were then incubated for another 3 hr at 37 °C. Subsequently, cell pellets were harvested and resuspended in 50 mM Tris pH 8.0, 100 mM NaCl, 5 mM beta-mercaptoethanol, 5 mM EDTA, 10 μg/mL DNaseI, and 1 mM PMSF. The lysate was sonicated and then clarified by centrifugation at 20,000 g for 45 min at 10 °C. The resulting insoluble material where histones are enriched was solubilized with 50 mM Tris-HCl pH 7.5, 1 M NaCl, 6 M Guanidine HCl, 0.1 mM PMSF, 0.5 mM TCEP, and 1% Triton X-100 and applied to a Ni-NTA agarose (Qiagen) or HisPur Ni-NTA (Thermo Fisher Scientific) resin for affinity purification. Ni resin was extensively washed with 50 mM Tris-HCl pH 7.5, 1 M NaCl, 6 M Guanidine HCl, and 0.5 mM TCEP and His_6_-tagged histones were eluted with the buffer supplemented with 300 mM Imidazole. Purified histones were concentrated with a centrifugal concentrator with a 10,000 MWCO, aliquoted and stored at −20 °C.

### Production of DNA for nucleosome reconstitution

The *Sc*GIT1 promoter DNA fragment for the assembly of nucleosome core particles was obtained by Gateway cloning (Thermo Fisher Scientific) and amplification in *E. coli*. The 147 bp DNA sequence upstream of the *GIT1* gene transcription start site corresponding to the genomic location 298,930 to 299,076 of the chromosome III in *S. cerevisiae* was selected for its strong nucleosome positioning characteristic in the yeast genome. To extract this *GIT1* DNA fragment from a bacterial plasmid, two EcoRV restriction sites were engineered at each site of the 147 bp *GIT1* DNA sequence, where EcoRV digestion left 3 bp segments at each side of the 147 *Sc*GIT1 DNA sequence resulting in a 153 bp DNA fragment competent for mono-nucleosome assembly. To clone this sequence, a dsDNA insert containing the 147 bp *Sc*GIT1 promoter sequence, the two EcoRV sites and Gateway recombination regions were synthetically manufactured (Integrated DNA Technologies) (Supplementary Table S9). The *Sc*GIT1 DNA insert was cloned into a Gateway pDONR221 vector devoid of EcoRV restriction sites on its backbone (pDONR221ΔEcoRV) followed by whole plasmid sequencing verification of the resulting plasmid (Supplementary Table S6).

The *Sc*GIT1 pDONR221ΔEcoRV plasmid was amplified in One Shot *ccd*B Survival 2 T1^R^ competent cells (Thermo Fisher Scientific) cultured in 2XYT media for 16–18 hr at 37 °C. Cells were harvested and the *Sc*GIT1 plasmid was extracted from cells by alkaline lysis and precipitated with isopropanol. Alcohol precipitated plasmid was resuspended in 10 mM Tris-HCl pH 8.0 and 50 mM EDTA. RNase was added to a final concentration of 20 μg/mL and incubated overnight at 37 °C. RNase-treated plasmid was then purified by polyethylene glycol (PEG) precipitation at 0.5 M NaCl and 10% (w/v) PEG 6,000 conditions. PEG precipitated *Sc*GIT1 pDONR221ΔEcoRV plasmid was collected by centrifugation and resuspended in nuclease-free water. The *Sc*GIT1 DNA fragment was extracted from the pDONR221ΔEcoRV plasmid backbone by EcoRV restriction enzyme digestion. For this, the plasmid was diluted to 2 mg/mL in 50 mM Potassium Acetate, 20 mM Tris-Acetate, 10 mM Magnesium Acetate and 100 μg/mL Bovine Serum Albumin (BSA) at pH 7.9 (CutSmart Buffer, New England Biolabs) and approximately 120 units of EcoRV-HF restriction enzyme (New England Biolabs) were added per nanomole of plasmid. The EcoRV digestion was conducted at 37 °C and incubated overnight. Complete digestion of the plasmid was determined by agarose gel electrophoresis. The excised 153 bp *Sc*GIT1 DNA fragment was purified from the rest of the pDONR221ΔEcoRV backbone by PEG precipitation at 0.5 M NaCl and 9% (w/v) PEG 6,000 conditions. PEG-precipitated plasmid backbone was removed from the solution by centrifugation and the 153 bp ScGIT1 DNA was subsequently precipitated by ethanol. Ethanol-precipitated *Sc*GIT1 DNA was resuspended in nuclease-free water and further purified with AMPure XP magnetic beads (Beckman Coulter). The final 153 bp *Sc*GIT1 DNA fragment was eluted from the magnetic beads in 10 mM Tris-HCl pH8.0 and 1 mM EDTA, and the concentration was determined spectroscopically by using the 260 nm extinction coefficient 2,987,200 M^−1^cm^− 1^. DNA was stored at −20 °C until use.

### Nucleosome core particle reconstitution

His_6_-tagged *Hs*H3.2, *Hs*H4, *Sc*H2B and wild-type or mutant *Sc*H2A.Z were combined at equimolar amounts in 50 mM Tris-HCl pH 7.5, 1 M NaCl, 6 M Guanidine HCl, and 0.5 mM TCEP at a final total histone concentration of ~ 0.067 mg/mL. To assemble and refold the histones into octamers, the histone mixture was dialyzed against 20 mM Tris-HCl pH 7.5, 2 M NaCl, 1 mM EDTA, 5 mM DTT and 0.5 M Guanidine HCl overnight at room temperature. His_6_-tags were removed from histones assembled into octamers by adding 2/100 histone-mass-equivalent of TEV protease and incubating overnight at room temperature. To assemble nucleosomes the 153 bp *Sc*GIT1 DNA was added at equimolar amounts to the cleaved octamer and the octamer-DNA mixture was dialyzed at room temperature against 10 mM Tris-HCl pH 8.0, 1 mM EDTA, and 5 mM DTT. Assembled nucleosomes were concentrated with a 10,000 MWCO centrifugal concentrator and stored at 4 °C.

### Thermal stability shift assay

Reconstituted H2A.Z nucleosomes were diluted at a final concentration of 1.134 μM in thermal stability assay buffer [20 mM Tris-HCl pH 7.5, 0.5 mM TCEP, and 0 mM or 150 mM NaCl]. The SYPRO orange dye (Sigma-Aldrich) was included in each sample at a final concentration of 5× in a 20 μL reaction volume. The fluorescence from the dye was monitored in a CFX Opus 384 real-time PCR system (Bio-Rad) from 25 °C to 95 °C with incremental steps of 1 °C. After each temperature was reached, the samples were incubated for 1 min prior to measurement of the fluorescence signal. At least three replicates for each nucleosome sample and negative control (only buffer) condition were prepared and measured. The raw fluorescence values of the SYPRO dye obtained from the real-time PCR machine (RFU) were normalized using the Eq. 1:

Eqn. 1 $$\text{normalized fluorescence}(\text{Ti})=\frac{{\text{RFU}}_{{\text{T}}_{\text{i}}}{-\text{RFU}}_{\text{min}}}{{\text{RFU}}_{\text{max}}{-\text{RFU}}_{\text{min}}}$$

Where RFU_Ti_ is the raw fluorescence at temperature i, RFU_min_ and RFU_max_ are the lowest and highest raw fluorescent values, respectively, obtained for each sample among the entire range of temperatures measured.

To better observe nucleosome denaturation transition steps, we plotted the rate of change of the normalized fluorescence signal (d(normalized fluorescence)/dT) as a function of temperature. The rate of change was calculated using the Eq. 2:

Eqn. 2 $$\frac{\text{d}(\text{normalized fluorescence})}{\text{dT}}=\frac{{\text{normalized fluorescence}}_{{\text{T}}_{\text{i}+1}}-{\text{normalized fluorescence}}_{{\text{T}}_{\text{i}}}}{{\text{T}}_{\text{i}+1}-{\text{T}}_{\text{i}}}$$

For plotting d(normalized fluorescence)/dT vs temperature, the resulting d(normalized fluorescence)/dT value was assigned to the Ti + 1 temperature. In this distribution, the temperature at each peak indicates the nucleosome melting temperature at nucleosome melting transition steps.

### Human cell lines and analysis

The H2A.Z-1 wild type or R31H mutant, and H2A.Z-2 wild type or L99R mutant cell lines were established by inserting 3×FLAG-2xStrep-tagged constructs in the *AAVS1* safe harbor locus in K562 cells as previously described[76], and clones with similar expression were selected and used for affinity purification, while whole cell extract and chromatin-enriched extract were performed directly on cell pools after 1 week of selection with puromycin post-transfection. Cells that integrated the empty 3×FLAG-2×Strep vector in the *AAVS1* locus were used as a control. K562 cells were obtained from the ATCC and maintained at 37°C under 5% CO_2_ in RPMI medium supplemented with 10% newborn calf serum (Wisent) and GlutaMAX (Gibco). Cells were transfected using the Amaxa Nucleofector (Lonza) per the manufacturer’s recommendations. Purification of tagged H2AZ.1 and H2AZ.2 was performed from 500 mL cultures basically as described[135]. Soluble nuclear extracts were prepared, adjusted to 0.1% Tween-20, and centrifuged at 100,000 g for 60 min. Extracts of roughly 1-1.5 mL were precleared with 150 μL Sepharose CL-6B (Sigma), then 60 μL anti-FLAG M2 affinity resin (Sigma) was added for 2 hr at 4°C. The beads were then washed in Poly-Prep columns (Bio-Rad) with 40 column volumes (CV) of buffer L + H300 (20 mM HEPES-KOH pH 7.9, 300 mM KCl, 1.5 mM MgCl_2_, 0.2 mM EDTA, 10% glycerol, 1 mM PMSF, 10 mM sodium butyrate, 10 mM b-glycerophosphate, 5 mM NaF, 0.1 mM Na_3_VO_4_, 2 μg/mL leupeptin, 2 μg/mL pepstatin A, and 2.5 μg/mL aprotinin), 40 CV of buffer Wash 1 (same as L + H300, but without MgCl_2_ and EDTA, however supplemented with 0.1% Tween-20 and 1 mM DTT ) and 40 CV of buffer Wash 2 (same as Wash 1, except 150 mM KCl). Two sequential elutions with 2.5 CV of Wash 2 buffer supplemented with 200 μg/mL 3x FLAG peptide (Sigma) were performed (1 hr at 4°C), and the eluted fractions were flash frozen in liquid nitrogen and stored at −80°C. The first elution was used for subsequent analysis by immunoblotting after normalization of the FLAG signal.

Chromatin enriched extract (CEE) extracts were prepared as followed: ~5 million cells were washed twice in 1× PBS and subsequently lysed in 300 μL cold ECB1 buffer (50 mM Tris pH 7.5, 100 mM NaCl, 0.5% NP-40, 1 mM EDTA, 1 mM DTT, 1 mM PMSF, 10 mM sodium butyrate, 10 mM b-glycerophosphate, 2 μg/mL leupeptin, 2 μg/mL pepstatin A, 2.5 μg/mL aprotinin, 1 uM Worthmannin, and 10 mM NEM ), centrifuged at 1,000 g for 5 min at 4°C, the pellet was washed once in 100 μL of ECB1 and solubilized at 30°C in 100 mL ECB2 (50 mM Tris pH 7.5, 300 mM NaCl, 5 mM CaCl_2_, 1 mM PMSF, 10 mM sodium butyrate,10 mM b-glycerophosphate, 2 μg/mL leupeptin, 2 μg/mL pepstatin A, 2.5 μg/mL aprotinin, and 0.04U/μL micrococcal nuclease ), centrifuged at 16,000g for 15 min at 4°C. Whole cell and chromatin enriched extracts (WCE and CEE), and affinity purified complexes were analyzed by immunoblotting using antibodies against H3 (Cat. ab17109, Abcam), FLAG M2-HRP (Cat. A8592, Sigma), BRD8 (Cat. A300-219A, Bethyl), DMAP1 (Cat. sc373949, Santa Cruz), Tubulin (Cat. CP06, Millipore), and YEATS4 (Cat. sc393708, Santa Cruz).

## Supplementary Material

This is a list of supplementary files associated with this preprint. Click to download.

• H2A.Zmanuscripttablesfinal.xlsx

• Supplementaryinformation.docx

## Figures and Tables

**Figurre 6:**
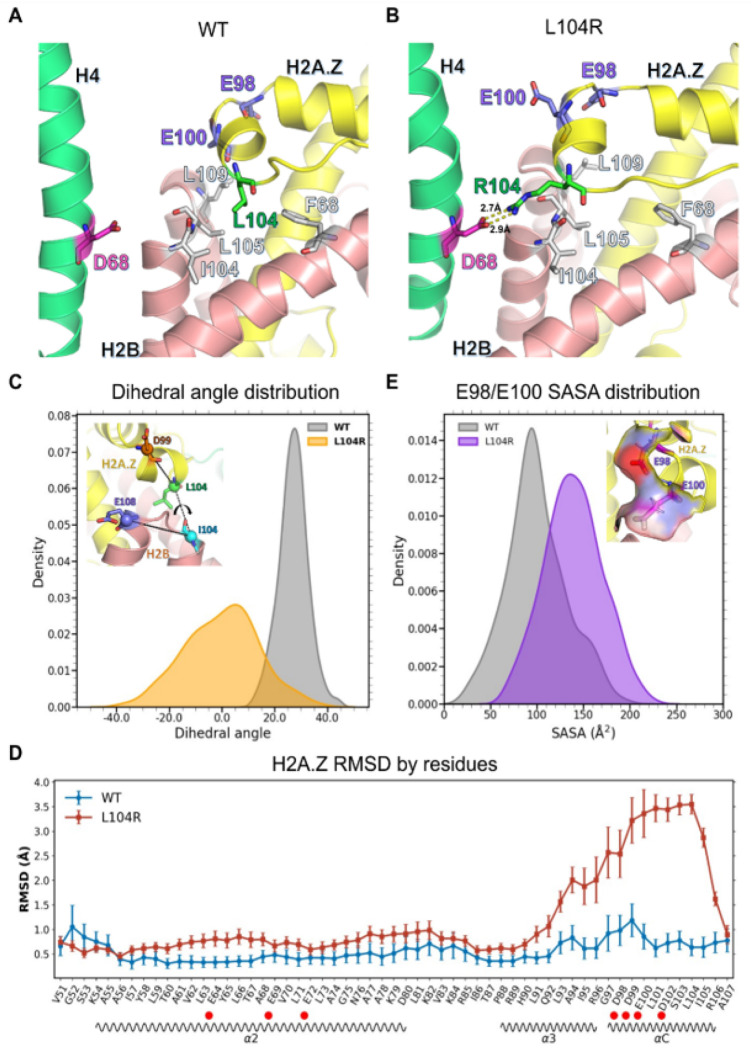
Atom-level mechanisms by which the L104R mutation influenced nucleosome structure **(A**) Representative conformation in wild-type nucleosomes showing H2A.Z L104 within the hydrophobic pocket formed by residues F68, I104, L105, and L109 on H2B. **(B)** Representative conformation in L104R showing H2A.Z R104 forming a salt bridge with H4 D68 and distorting the H2A.Z αC helix. **(C**) Comparison of the D99-L104-I104-I104 dihedral angle (θ) distributions between wild type (grey) and L104R mutant (orange). **(D)** Comparison of average RMSD values of H2A.Z (relative to initial conformations) between wild type (blue) and the L104R mutant (red). Red circles show the H2A.Z acidic patch residues. **(E)** Comparison of SASA distributions of H2A.Z acidic patch residues E98 and E100 in wild type and the L104R mutant.

## Data Availability

Strains are available upon request. ChIP-seq data is available at ENA at MBL-EBI under accession number ERP183505 (https://www.ebi.ac.uk/ena/browser/view/ERP183505).
